# “We just have to help”: Community health workers' informal task-shifting and task-sharing practices for hypertension and diabetes care in Nigeria

**DOI:** 10.3389/fpubh.2023.1038062

**Published:** 2023-01-26

**Authors:** Whenayon Simeon Ajisegiri, Seye Abimbola, Azeb Gebresilassie Tesema, Olumuyiwa O. Odusanya, David Peiris, Rohina Joshi

**Affiliations:** ^1^The George Institute for Global Health, University of New South Wales (UNSW), Sydney, NSW, Australia; ^2^School of Public Health, University of Sydney, Sydney, NSW, Australia; ^3^School of Public Health, Mekelle University, Mekelle, Ethiopia; ^4^Department of Community Health and Primary Health Care, Lagos State University College of Medicine, Ikeja, Nigeria; ^5^School of Population Health, University of New South Wales (UNSW), Sydney, NSW, Australia; ^6^The George Institute for Global Health, New Delhi, India

**Keywords:** community health workers, primary health care, service delivery, hypertension, diabetes, non-communicable diseases, skills

## Abstract

**Introduction:**

Nigeria's skilled health professional health workforce density is insufficient to achieve its national targets for non-communicable diseases (NCD) which include 25% reduction in the prevalence of diabetes and hypertension, particularly at the primary health care (PHC) level. This places a great demand on community health workers (CHWs) who constitute the majority of PHC workers. Traditionally, CHWs are mainly involved in infectious diseases programmes, and maternal and child health services. Their involvement with prevention and control of NCDs has been minimal. With government prioritization of PHC for combating the rising NCD burden, strengthening CHWs' skills and competencies for NCD care delivery is crucial.

**Methods:**

We conducted a mixed methods study to explore the roles and practices of CHWs in the delivery of hypertension and diabetes care at PHC facilities in four states (two each in northern and southern regions) in Nigeria. We reviewed the National Standing Orders that guide CHWs' practices at the PHC facilities and administered a survey to 76 CHWs and conducted 13 focus groups (90 participants), and in-depth individual interviews with 13 CHWs and 7 other local and state government stakeholders.

**Results:**

Overall, we found that despite capacity constraints, CHWs frequently delivered services beyond the scope of practice stipulated in the National Standing Orders. Such informal task-shifting practices were primarily motivated by a need to serve the community.

**Discussion:**

While these practices may partially support health system functions and address unmet need, they may also lead to variable care quality and safety. Several factors could mitigate these adverse impacts and strengthen CHW roles in the health system. These include a stronger enabling policy environment to support NCD task-sharing, investment in continuous capacity building for CHWs, improved guidelines that can be implemented at the point of care, and improved coordination processes between PHC and higher-level facilities.

## Introduction

Most countries have a critical shortage of skilled health workers, in particular doctors and nurses (1, 2). The World Health Organization (WHO) recommends a skilled health professional density of at least 4.45/1,000 population to achieve the sustainable development goals (SDGs) (3). The majority of countries in Africa have densities <2.28/1,000 population (4). Despite having one of the largest health workforces on the continent, Nigeria's skilled health workforce density is only 1.95/1,000 population, well below the recommended benchmark (5). Health system weaknesses due to inadequate number and skills of health workers is most pronounced at the primary health care (PHC) level in Nigeria. These weaknesses threaten the achievement of Universal Health Coverage and national NCD targets which includes about 25% reduction in the premature death from NCDs, prevalence of diabetes, hypertension and obesity (6);—and the ability to address the growing burden of non-communicable diseases (NCDs) in Nigeria.

The deployment of community health workers CHWs to deliver essential health services has proven to be a well-established strategy to address critical skilled health workforce (7, 8). The term CHW is a broad category comprising and although there are over 30 designations different types of health cadres globally, they share the common role of working at the frontline in community (9). The WHO considers CHWs to be members of the community with varying levels of formal education who are trained to address the health problems of individuals and the community (10). They usually share similar life experiences, socio-economic status and ethnicity with the communities they serve (11). CHWs often form the backbone of most health systems (11) at the PHC level and provide a linkage between communities and health systems (12).

The diversity in CHW roles is driven by a wide variation in the duration, content, and level of their training. While some CHWs are formally trained according to structured curricula and have a specified duration in government-recognized institutions, others are informally trained with access to variable content and may work in unregulated settings (13). Although CHW is a generic term used in most countries, each country usually has specific terms and scope of practice appropriate for their CHW cadre. For instance, CHWs are called Accredited Social Health Activists and auxiliary nurse midwives in India (14), Health Extension Workers (HEW) in Ethiopia (15), and Family Welfare Assistants or health assistants in Bangladesh (16).

In Nigeria, CHW titles include Community Health Officers (CHOs), Community Health Extension Workers (CHEW) and Junior Community Health Extension Workers (JCHEW) who have received various degrees of training at government-recognized institutions (6). JCHEWs and CHEWs hold Certificates and Diplomas in community health after completing 2- and 3-year training programmes respectively at Schools of Health Technology. CHOs are CHEWs who possess a higher national diploma obtained through an additional 1-year training programme in PHC at a teaching hospital (17).

The roles and responsibilities of CHWs in Nigeria depend on factors that include (but are not limited to) the level of education, type of training received, health care setting, size of population serviced and geographical coverage (18). These roles may be general or specialized (19) and span health promotion, prevention and treatment of diseases as well as management of data (8). The practice of CHWs is regulated by National Standing Orders which are the primary guidelines for CHW training and delivery of services to the community. They typically describe clinical features of common disease conditions and how they should be managed and define the scope of CHW practice under the instruction and supervision of physicians (20). Apart from ensuring normative guidance in the quality of care, adherence to the National Standing Orders also offer legal protection to CHWs in the course of service provision (21, 22).

CHWs have been traditionally involved in programmes that target infectious diseases and maternal and child services. Their involvement in the prevention and control of NCDs is relatively new and tends to be less well-documented (23). Although some studies have assessed the effectiveness of CHWs in the prevention and management of NCDs elsewhere (24, 25), there is a dearth of studies on CHWs' engagement in NCD care in Nigeria. One study on CHW management of hypertension and diabetes identified substantial knowledge gaps in the diagnosis and treatment of these NCDs (26). A pilot study to assess CHW support and self-home blood pressure (BP) monitoring found that such CHW support had potential to be successfully implemented in PHC settings in Nigeria (27). A study that explored stakeholders' perspectives on the adaptation of a hypertension treatment program for PHC facilities in Nigeria suggested empowering CHWs through training to participate in team-based care was a major enabler to hypertension treatment in those facilities (28).

Given the current knowledge gaps and the central role played by CHWs in the PHC workforce, an assessment of their skills and competencies for carrying out NCD-related activities is crucial to develop and implement NCD policies and programmes in Nigeria. Because the National Standing Orders do not address service delivery for cancers and chronic respiratory illnesses, we focused on hypertension and diabetes care. Study aims were to: (1) describe the role of CHWs in the prevention and control hypertension and diabetes in PHC facilities in Nigeria; (2) identify the policies and practice gaps by comparing CHWs' actual practices with those stipulated in their guidelines; and (3) highlight opportunities for enhancing CHW support to address those gaps.

## Methods

We conducted a cross-sectional study with mixed methods data collection approach that included the following: (1) analysis of policies and guidelines related to CHW practices for hypertension and diabetes service delivery; (2) a cross-sectional survey of CHWs to quantify actual NCD service delivery practices; and (3) key informant interviews (KII) and focus group discussions (FGDs) with CHWs and other stakeholders to understand the processes involved in the reported practices in the survey.

### Study setting

The study was conducted in 13 PHC facilities across four states in Nigeria (two states in each of the northern and southern regions) between July and September 2019. The PHC facilities were purposively selected to obtain a diverse sample based on available human resources and health-seeking behaviors across the northern and southern regions of the country. This resulted in three PHCs selected per state except in one state where four PHCs were selected.

### Data collection

We started by reviewing and summarizing the National Standing Orders for hypertension and diabetes control for JCHEWs, CHOs and CHEWs, and PHC guidelines in Nigeria (21, 22, 29).

An interviewer-administered survey was used for obtaining information from CHWs on their socio-demographic characteristics and service provision (overall and specific to NCD-related activities). The survey included elements adapted from the WHO Package of Essential NCDs (WHO PEN) intervention (30) and the United States Agency for International Development Community Health Workers Assessment and Improvement Matrix (CHWAIM) toolkit (31) (Supplementary Appendix 1). CHWAIM was developed in 2011 to help government and non-governmental organizations assess, improve and plan CHW programmes and address implementation gaps. Although it has a maternal, newborn and child health and infectious diseases focus, it is designed to be adaptable for other services (31). In 2018, the Programme Functionality Matrix of the CHWAIM toolkit was reviewed and updated through a systematic review and extensive stakeholder consultation, resulting in ten criteria (32). We adapted these ten criteria or domains to understand the role of CHWs in their workplace, particularly in providing NCD care (Box 1).

Box 1 **CHWAIM survey domains.**

i. **Role and recruitment:** How are CHWs recruited and how are their roles defined?
ii. **Training:** What pre-service and in-service training is provided to CHWs to provide quality care and how are they evaluated?
iii. **Accreditation**: How is knowledge assessed and accredited during pre-service and in-service?
iv. **Equipment and supplies**: What is the availability and access to the required equipment?
v. **Supervision**: How is supportive supervision conducted?
vi. **Incentives**: How are CHWs incentivized/remunerated (both financial and non-financial incentives)?
vii. **Community involvement**: What is the level of community involvement in the CHWs' programmes?
viii. **Opportunity for advancement**: Is career progression an option available to the CHWs?
ix. **Data**: How does data flow from and to the community?
x. **Linkages to the national health system**: What policies are in place that integrate and include CHWs in health system planning, budget, and logistical support?

We then used findings from the survey to develop CHW interview guides. All three cadres of CHWs across 13 PHC facilities were invited to participate (JCHEWs, CHEWs, and CHOs). Sampling of CHWs for interview participation was not stratified by category, rather an invitation was sent out to all CHWs. FGDs were conducted at each PHC facility, and KIIs were conducted with the head or most senior CHW in a facility. We also interviewed state and local governments stakeholders to understand their perspectives on CHWs' NCD-related work in PHC facilities. These stakeholders supervised CHWs and are involved with disease control (including NCDs) activities at the state and local government levels. Interviews were conducted by the lead author (WSA), who has worked with the Nigerian government at national and frontline level of the country's health system. He was supported by two other trained data collectors. Each FGD involved 5–10 participants, lasting 45–75 min. Each KII lasted 30–45 min. The interviews (KII and IDIs) and FGDs were conducted in English language, digitally recorded and transcribed verbatim.

### Data analysis

Survey responses were tabulated using Microsoft Excel. Descriptive variables were illustrated as frequencies and proportions. Qualitative data were coded in NVivo Pro 12. Initial themes were guided by the survey findings and focused on understanding the gaps between policy, guidelines and practice. The research team met regularly to analyses and interpret the themes emerging from the interviews. These meetings helped to refine themes, make appropriate inferences and synthesize findings across study sites.

### Ethical considerations

Ethical approval was granted by the National Health Research Ethics Committee of Nigeria (Approval no: NHREC/01/01/2007) and the University of New South Wales Human Research Ethics Committee (HC: 190051). Informed written consent was obtained from all participants that contributed data to the study. Anonymity and confidentiality of all respondents was maintained throughout, and participants names were replaced with codes during data analysis and reporting.

## Results

**Figure 1:**
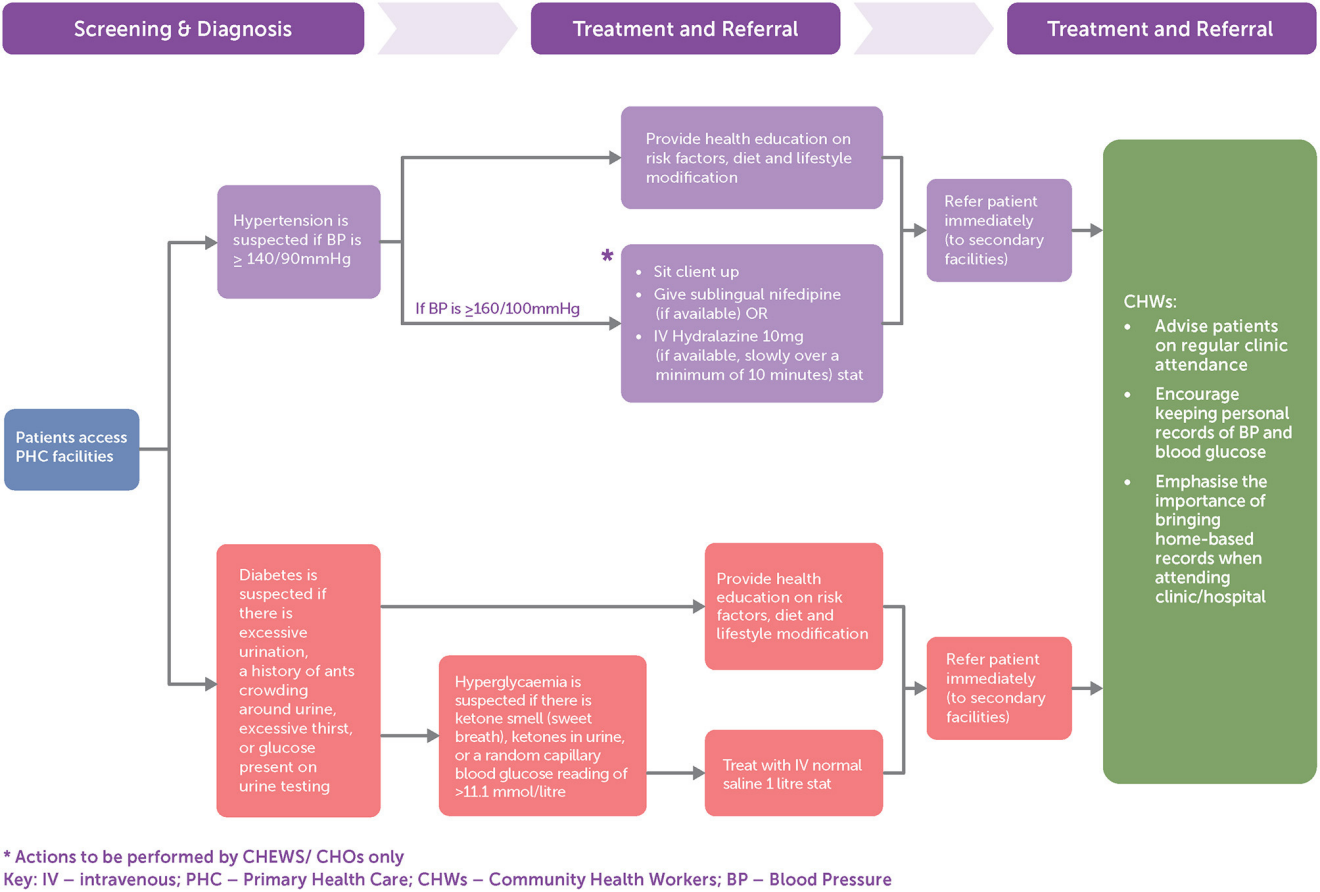
Care of patients with hypertension and diabetes according to National Standing Orders for Junior Community Health Extension Workers (JCHEWs), Community Health Extension Workers (CHEWs), and Community Health Officers (CHOs).

Figure 1 summarizes the National Standing Orders care pathways for CHWs in the delivery of care for hypertension and diabetes at PHC facilities. Management guidance is focused on acute care for people presenting either with elevated blood pressure or symptoms that may be associated with diabetes. There is minimal focus on preventive activities or ongoing chronic care once a diagnosis of hypertension or diabetes is made. This includes limited guidance on: (1) preventive screening of risk factors for diabetes or hypertension; (2) ongoing risk factor monitoring for prevention of complications in patients with established diabetes or hypertension, (3) assessment of cardiovascular disease risks by the CHWs; and (4) follow up treatment with feedback from referral centers (to ensure continuity of care).

### Survey findings

Among the 77 CHWs (CHO−9, CHEW−53, JCHEW−15) who participated in the survey on NCD care (representing a response rate of 85%), the vast majority were female, [70 (91%)] with a mix of full-time employment [36 (47%)] and volunteer contract [41 (53%)] workers. The majority worked in a facility with at least one nurse, however a minority worked with a doctor (Table 1). Although the majority reported receiving supervision in some areas, a substantial proportion reported receiving no supervisory support at all. Despite high levels of job satisfaction, many CHWs reported barriers to remaining in the job, particularly because of remuneration and in adequate administrative and health professional support.

**Table 1:** Characteristics of participants (CHWs) (*n* = 77).

**Socio-demographic variables**	**Frequency (proportion) *n* (%)**
**Age (years)**	
≤ 29	13 (17)
30–39	25 (33)
40–49	32 (42)
50–59	7 (9)
***Mean age (SD):*** 37.5 years (±8.9)	
**Sex**	
Female	70 (91)
Male	7 (9)
**Marital status**	
Married	69 (79)
Unmarried	8 (21)
**Categories of CHWs**	
Community Health Officers	9 (12)
Community Health Extension Workers (CHEWs)	53 (69)
Junior Community Health Extension Workers (JCHEWs)	15 (19)
**Employment type**	
Full-time	36 (47)
Volunteer/contract	41 (53)
**Highest education level**	
Basic national diploma	49 64
Higher level national diploma	17 (22)
Bachelor or master's degree	11 (14)
**Duration of working as a CHW**	
<3 years	5 (7)
3–10 years	38 (49)
>10 years	34 (44)
**Nurse and doctor availability at the facility**	
≥1 nurse	56 (72)
≥1 doctor	28 (36)
**Type of supervision received**	
Direct observation of service delivery	61 (79)
Coaching and skills development	53 (69)
Problem solving	56 (73)
Health record review	55 (71)
Equipment supply check	46 (60)
No supervision received at all	12 (16)
**CHW satisfaction**	
Satisfied overall with being a CHW	70 (91)
Intend to continue working as a CHW in the future	68 (88)
**Reasons CHWs may consider leaving their job**	
Inadequate salary	46 (60)
Low support from the higher administrative bodies	43 (55)
Not respected or recognized by other health workers	42 (55)
Not respected by the community	22 (29)
Excess workload	18 (23)
Family or personal reasons	31 (40)

Table 2 illustrates the self-reported services provided by CHWs compared with the in-service training received for conducting such activities. Blood pressure monitoring was the commonest activity conducted (88%) while cancer screening was the least (17%). CHWs engaged in more activities than they were formally trained for (except for awareness-raising activities for tobacco cessation), and the gap between conducting an activity and receiving formal training ranged from 8 to 31%.

**Table 2:** NCD-related activities carried out by CHWs.

**Domain**	**Activities**	**Routinely conducted *N* (%) (A)**	**Received formal training (%) (B)**	**Gap between conducting activity and training (A–B)%**
Registration of basic demographic and clinical data	Register adults with HTN	53 (69)	22 (33)	31
	Register adults with diabetes	38 (49)	23 (30)	19
Screening/early identification of people with NCDs	Organize screening for HTN	37 (48)	21 (27)	21
	Organize screening for diabetes	34 (44)	22 (29)	15
	Organize screening for cancers	13 (17)	5 (7)	10
Increasing community awareness through engagement and mobilization	Community awareness on fruits and vegetable	59 (77)	44 (57)	20
	Community awareness on physical activities	52 (68)	39 (51)	17
	Community awareness on salt intake reduction	50 (65)	36 (47)	18
	Community awareness on tobacco cessation	41 (53)	54 (71)	−18
	Community awareness on weight control	53 (69)	35 (46)	23
Intervention for patients with NCDs	Regular home visits for NCDs patient (to encourage continuity of care)	46 (60)	34 (44)	16
	Follow up to ensure medication adherence	49 (64)	39 (51)	13
	BP measurement to monitor HTN patients	65 (88)	51 (66)	22
	Blood glucose measurement for DM patients	38 (49)	27 (35)	14
	Counseling patients on smoking cessation	51 (66)	33 (43)	23
	Counseling patients on adopting a healthy diet	54 (70)	33 (43)	27
	Counseling patients on weight control	53 (69)	33 (43)	26
Referral system	Counseling and motivation for referral	59 (77)	40 (52)	25
	Mobilizing support from community to effect referral	44 (57)	31 (40)	17
	Effecting referrals for all cases to the next level	55 (71)	37 (48)	23
	Accompanying patients to next level HF	47 (61)	38 (49)	12
	Get feedback and follow up referred patients	53 (69)	38 (49)	20
Essential drugs	Replenishment of essential drugs at the facility	23 (30)	17 (22)	8
	Prescribed drugs for HTN or DM patients	34 (44)	27 (35)	9
	Refill drugs for HTN or DM patients	34 (44)	17 (22)	22

CHWs identified inadequate training (84%), inadequate supplies of equipment (81%), poor infrastructure (71%) and inadequate supervision (52%) as the most frequent barriers to delivery of hypertension and diabetes care.

### Qualitative findings

In total, 13 FGDs, 13 KII interviews with facility heads and 7 KIIs with other stakeholders were conducted (Table 3).

**Table 3:** Interview and survey participants.

**Community health workers**			
**Facility/region**	**KII participants**	**FGD participants**	**Survey participants**
PHC facility 1, North	KII1: M, 54 years, CHEW	FGD1: 4 M, 6 F	7
PHC facility 2, North	KII2: F, 40 years, CHO	FGD2: 5 F	4
PHC facility 3, North	KII3: F, 45 years, CHO	FGD3: 3 M, 6 F	5
PHC facility 4, North	KII4: F, 45 years, CHEW	FGD4: 2 M, 6 F	7
PHC facility 5, North	KII5: F, 49 years, CHO	FGD5: 1 M, 5 F	6
PHC facility 6, North	KII6: F, 55 years, CHO	FGD6: 4 M, 6 F	9
PHC facility 7, North	KII7: F, 48, years CHO	FGD7: 2 M, 4 F	6
PHC facility 8, South	KII8: M, 55 years, CHO	FGD8: 4 F, 6 F	7
PHC facility 9, South	KII9: M, 55 years, CHO	FGD9: 3 F, 6 F	6
PHC facility 10, South	KII10: F, 40 years, CHO	FGD10: 10 F	5
PHC facility 11, South	No participant	FGD11: 10 F	4
PHC facility 12, South	KII12: F, 38 years, CHO	FGD12: 8 F	5
PHC facility 13, South	KII13: F, 41 years, CHO	FGD13: 6 F	6
**Stakeholders**			
**Agency/organization**	**Designation**		
State ministry of health	KII-S1: male, director of public health		
State primary health development agency	KII-S2: male, director of health planning research and statistics		
	KII-S3: male, director of disease control		
	KII-S4: male, director, community health services and education		
	KII-S5: male, medical officer of health		
	KII-S6: male, medical officer of health		
Community health practitioner registration board of Nigeria/college of health technology	KII-S7: male, senior lecturer		

Three major themes were identified which influence how CHWs provide hypertension and diabetes care. These related to: (1) variable implementation of the National Standing Orders; (2) CHW role expansion and informal task-shifting; and (3) weak referral linkages.

### Variable implementation of National Standing Orders

Despite the National Standing Orders being the main guidance for management of hypertension and diabetes, the CHWs interviewed appeared to interpret them in varying ways. For instance, there was a wide interpretation both between and within facilities of what constituted hypertension “*….If it is above normal, like 140/100 [mmHg]..”* ***(KII7)……,*** *or “…If somebody has, let's say, he has 170/100 [mmHg], or if he has [systolic of] 140 or 150 [mmHg]….”**(FGD9 participants). and*** “*....when we take their BP, when the BP is high, maybe its 150 over 90 [mmHg]…”* ***(FGD7 participants.)*** Similarly for assessing diabetes, some CHWs were familiar with some clinical features but there was less familiarity with interpreting blood glucose values and criteria for diagnosing a patient with diabetes “*…because we are meant to understand that [blood sugar] between 3.5 and 5.9 mmol/l is normal, then from this [value] upward is diabetes…”* ***(FGD4*** ***participants)***.

Another limitation to implementation of the National Standing Orders was medication and equipment availability. This led to CHWs having to “improvise” to assess blood pressure and blood glucose: “*Under normal circumstances, different sections or units [are] supposed to have all those equipment, but [when] one section has to wait for the other to finish ….. we design a modality … where all patients that come in [check] their vital signs [centrally] before they go to wherever they are going to. So, [because] there is no equipment, we're just improvising”* ***(FGD13 participants)***. This is particularly a problem for blood glucose testing where patients may be charged a fee for testing and not be able to pay, or glucometers are only available for part of the day: “*….. the glucometer is stationed in the lab, and it doesn't run 24 h, so during the night shift, if you have such cases, … you can't help [the patient]…*.” **(FGD4 participants)**.

### CHW role expansion and informal task-shifting

Although the National Standing Orders recommend that CHWs refer patients suspected or diagnosed with hypertension, many CHWs were comfortable to initiate treatment and provide ongoing medication management: “*…some will have 130/80, or 140/80, we just give..and ask the person to have rest or come back the second day, to recheck. But, if it is more than that, we can give nifedipine or amlodipine. So, those are the drugs that we use to prescribe* ***(KII9)***. Other CHWs would initiate management and then refer if there was a perceived failure to achieve control: “*…Like if a patient comes in with BP*<*160/100[mmHg], we try to manage between 23 days, if it doesn't come down you have to refer because there might be something ….”* ***(KII4)***. Similar care processes were described for patients with diabetes“*…if the blood sugar is high,….. we may place the patient on daonil [glibenclamide], just for maybe 3 or 5 days. …..Then, and we advise the patient on diet…..”* ***(FGD13*** ***participants)***.

The criteria for initiating and continuing treatment varied across facilities, based on the attending CHW's discretion and medication availability within the PHC facility. One focus group participant referred to “mild” forms of medication to initiate treatment “*.…It is the CHEW that will give the prescription, and in that aspect, we give the mild one, like nifedipine, amlodipine and diuretic…. we don't give the higher one because, you are trying to just initiate the patient……”* ***(FGD1 participants)***.

Although CHWs exercise considerable discretion in how they implement the National Standing Orders, by contrast several government stakeholders held more rigid views that their role should be restricted to screening and referral. Many felt that the formal training provided to CHWs in the college of health technology is insufficient for taking on a treatment role: “*….. the reason why they are training them is so that they can recognize (non-communicable diseases) …. if they see anyone with it they can refer…”* ***(KII- Stakeholder)***. Another justification for restricting CHW scope of practice was related to limited medication supply. Although NCD drugs are on the list of essential drugs and should be available at PHC facilities, frequently such medications are not available: “*… since they (CHWs) are not treating it, they don't need to buy..….. there are no drugs for NCDs in the PHC* ***(KII-*** ***Stakeholder)***.

One motivator for extending their scope of practice beyond that stipulated in the National Standing Orders' is the sense of addressing unmet need: “*….. where our “Standing Orders” say you should treat hypertension, most…will say “refer”, …(but) by initiating or starting them on medication, … we are just helping….”* ***(FGD1 participants)***. Some government stakeholders felt an expanded scope of practice was needed to maintain patients' trust in the services provided at the PHC facility. Because referring patients to higher level facilities could negatively affect perceptions of PHC level care, participants believed that CHWs had to strike a balance “…*so that it doesn't water down the respect they have for the [PHC] system…. If you refer, the person needs to understand that it's not because of the fact that you are not competent to care … In terms of NCDs also, if we don't manage that aspect carefully, it may also affect patronage…”* ***(KII-*** ***Stakeholder)***.

Another important motivation was the sense of prestige derived from displaying similar skills to doctors. Some CHWs believed that they've worked long enough with physicians to acquire the experience needed to treat patients: …. “*we have worked longed enough with doctors, so, we also have the experience [to treat hypertension]…* ***(FGD5 participants)***.” This appears to be influenced by gender with one stakeholder commenting that male CHWs functioned like doctors at the PHC facility: “*…in the past, there were no doctors at the PHC system…. Every male you see is “a doctor”… what doctors are doing now, were being handled by CHOs …. they do prescribe anti-hypertensive and anti-diabetics [but] the extent to which they can is something to debate”* ***(KII-*** ***Stakeholder)***.

### Weak referral linkages and non-compliance with referral guidelines

A related barrier to optimal care was the perception that referral processes are inadequate. CHWs use a two-way referral form which is intended to support communication between CHWs at PHC facilities and health workers at secondary health facilities. Implicit in this is the expectation that the secondary facility staff will refer patients back to PHC facilities with clear feedback to aid continuity of care. CHWs commented that this feedback provides a learning opportunity on “*what to do next when there's another patient with a similar case…”* ***(FGD13 participants)***. However, many participants said the feedback from secondary health facilities is often not given or ineffective: “*…. after referring, most of these facilities don't give a response back. We are the only ones that attempt to call the patient to know their well-being.”* ***(FGD13 participants)***. The lack of feedback may result in CHWs initiating a new treatment plan at their own discretion“*…since I've been working here, I've not seen any feedback. …. That is why we do make calls to contact the patient…. It's very, very important [to get feedback].. we will know the health of that patient… but since we are not getting feedback, there's nothing we can do…”* ***(KII13)***. As a strategy to overcome the challenges of feedback from secondary facilities, some CHWs employed workarounds such as directly telephoning or visiting secondary health facilities. Unfortunately, such a resource intensive effort such as this would not address the problem: “*We normally go there to collect the feedback ourselves, and sometimes when we go there we do not even get the feedback….they will say the doctor is yet to sign it, the patient will be well, and the feedback is not yet signed* ***(KII6)***.

## Discussion

This mixed methods study explored practices of CHWs in hypertension and diabetes care in two states in Nigeria. We found that CHWs flexibly implement national guidelines for hypertension and diabetes exercising considerable discretion in interpretation. Further, we identified considerable workforce capacity gaps, system barriers including inadequate medication and equipment supplies, and challenging referral processes characterized by limited communication between PHC facilities and higher-level facilities. The findings suggest the need for a re-appraisal for how NCDs are integrated into PHC care with consideration of the following four areas: (1) formalize task-sharing and task-shifting policies for NCDs among CHWs; (2) enhance the National Standing Orders with simplified NCD clinical algorithms/guidelines that can easily be used by CHWs at the point of care; (3) Provide continuous capacity building for CHWs to enhance their roles in NCD care; and (4) improve care coordination between PHC and higher-level facilities.

### Formalize task-sharing and task-shifting policies for NCDs among CHWs

CHWs are central to shifting or sharing tasks traditionally undertaken by skilled health workers (33). Although task shifting models involving CHWs have been successfully implemented in Nigeria for maternal and child health programmes, infectious diseases management and provision of contraceptive implants (34, 35), such models have thus far been overlooked in NCD service delivery. Despite the lack of explicit policies in this area, we found abundant evidence of CHWs informally taking on the roles of physicians. Given the widespread nature of such informal task-sharing care models, it would be short-sighted and impractical to eliminate such practices. Rather, such practices may need to be explicitly endorsed, formalized into policy and supported by the health system—all requiring a substantial shift in what is currently stipulated in guidelines and policies.

In settings where health system pressures from NCDs are growing, task-shifting and task-sharing models of service delivery by non-physicians play a central role (36). There is mounting evidence that such strategies are feasible and cost-effective in LMICs (37, 38). However, this requires considerable strengthening and restructuring of the PHC system as task-shifting functions cannot occur in isolation and wider system reforms are required (38, 39). It requires appropriate remuneration structures, enhanced commitment to capacity building, provision of supportive supervision and active engagement with physicians, development of workforce policies that support team-based care, and the creation of the appropriate environment for its implementation, including addressing complex challenges such as role overlaps between different health worker cadres and community perceptions that might impact demand-side factors. Nigeria has considerable experience in implementing task-shifting structures for HIV and reproductive health and this should be leveraged for including reforms for NCD care (34, 35). In the absence of such reforms, informal practices will remain tacit, of variable quality and with high potential for unsafe care.

### Enhance CHW standing orders with simplified NCD clinical algorithm/guidelines that can be used at the point of care

Although the National Standing Orders are intended to guide CHW practices, they lack sufficient clarity to ensure compliance. The structure and volume of the Standing Orders themselves may pose a challenge for adherence and there is a need to have more simplified, unambiguous, NCD-specific clinical algorithms that are easy to use at the point of care. The current National Standing Orders do not include cardiovascular risk assessment as recommended in WHO guidelines. By adopting such an approach, CHWs will have greater guidance on implementation of a total risk approach to care leading to improved identification of people most in need of referral and treatment (30). There is robust evidence that such an approach can be feasibly and effectively implemented in many LMICs (40, 41), especially when the services are being delivered by non-physicians (42). Digital clinical decision support tools have also been shown to support task sharing models for hypertension and diabetes care (43–46). Currently there is little work being undertaken in this area in Nigeria. Such algorithms also need to be accompanied by appropriate and regular supportive supervision and accountability to support their uptake. There is also a need to integrate such supervision into existing training processes for infectious and other diseases rather than establishing separate structures.

### Provide continuous capacity building for CHWs

The wide discretion with which CHWs engage in care practices combined with minimal training opportunities was a key study finding. These practices are indications of system weakness and suggest high levels of unmet need that CHWs are attempting to address (47). While CHWs were motivated by multiple factors to act beyond their scope of practice in the delivery of care for NCDs, ensuring a functional PHC facility and the need to provide care for their communities appeared to be the dominant motivations. This sense of duty may be accompanied by an increase in perceived professional status. Some CHWs felt empowered when members of their community viewed them as integral members of the health system (48), comparable to other professionals such as nurses and doctors (49). As CHWs are strongly motivated to strengthen the health system for the communities they serve (50), there are major opportunities to further empower them, commensurate with their desired competencies. This would support them to provide an optimum quality of care and mitigate against feelings of being undervalued or undermined in their communities and facilities of practice. Empowerment of CHWs to strengthen NCD service delivery requires multi-level capacity building at substantially greater degree than what is currently being provided. Such capacity building needs to be implemented in training colleges and then continuously supported with pre-service and regular in-service training (41, 51).

### Improve care coordination between PHC and higher-level facilities

To support follow up and long-term monitoring of patients with hypertension and diabetes by CHWs at the PHC facilities, this study identified the need to strengthen the referral and feedback processes and enhance care continuity and quality (52, 53). This can be partly achieved by making simplified referral guidelines available. As seen in this study and others, non-adherence to referral guidelines was common practice among CHWs (54, 55). It is therefore important to implement more effective mechanisms to support adherence to referral guidelines including supportive supervision and continuous CHW training (56). Although these findings are limited to NCDs (in particular hypertension and diabetes management), they are likely to be relevant to other areas of CHW practice.

However, there are also important discrepancies in perceptions of CHW roles, their scope of practice, and the functions of the PHC sectors more broadly that must also be addressed. These discrepancies may represent a deeper issue of how CHW roles and responsibilities are defined in policy, while they themselves do not have significant input into defining their own practice (57). The insights and skills that CHWs might have developed in the course of overcoming challenges in their practice are often not recognized when new policies and initiatives are developed (58). English and colleagues highlight that the tacit and contextual knowledge held by those in practice must be harnessed for implementing successful interventions (59). The omission of frontline worker perspectives could undermine success of health system reforms (60). These issues highlight the need to support PHC teams in which health workers of disparate training levels can work together to deliver accessible, high quality, coordinated care whether that be in PHC or higher-level care facilities (61).

### Study limitations

The findings of this study may not hold across all PHC facilities in Nigeria, especially those PHC facilities with physicians fully on staff. However, the majority of the PHC facilities have no or limited physician presence. Another possible limitation of the study is that the findings could be influenced by courtesy bias, particularly given we relied on self-reported data to determine care practices. To minimize these biases, we assured the participants of strict confidentiality and the potential benefits to improving workforce support if the authentic situation was presented. We also were able to triangulate the findings across the quantitative and qualitative data sources. Lastly, our study did not directly explore community and patient perspectives with respect to NCDs care. This could provide a deeper understanding of demand-side barriers to NCD care including perceptions of the role of CHWs, and identify opportunities for greater community engagement to support improved implementation of NCD policies. We recommend that future studies should explore this aspect.

## Conclusion

CHWs play key roles at the PHC level in addressing the growing burden of NCDs in Nigeria. However, these roles are at times beyond their allowed scope of practice and greatly limited by access to adequate training and supervision. This could compromise health care quality, raising the need to better equip this workforce for effective NCD service delivery at PHC facilities in Nigeria. Such considerations include formalizing task-sharing and task-shifting policies for NCDs among CHWs and fostering an enabling environment for their implementation; enhancing the National Standing Orders Development with point of care clinical algorithm/guidelines; continuous capacity building during working life; improved care coordination processes between PHC and higher-level facilities; and the promotion of multi-disciplinary team based approaches. Development of new policies in these areas should include substantial engagement with CHWs in their design. CHWs are highly motivated to deliver high quality NCD services and there are major opportunities to better support and leverage this workforce to strengthen Nigeria's health system response to NCDs.

## Data availability statement

The raw data supporting the conclusions of this article will be made available by the authors, without undue reservation.

## Ethics statement

The studies involving human participants were reviewed and approved by National Health Research Ethics Committee of Nigeria (Approval no: NHREC/01/01/2007) and University of New South Wales Human Research Ethics Committee (HC: 190051). The patients/participants provided their written informed consent to participate in this study.

## Author contributions

The study was conceptualized by WA, RJ, and DP. Data collection was conducted by WA and supported by OO. WA, RJ, OO, DP, and SA were involved analysis of the qualitative data. All authors provided critical intellectual input during the analysis. Manuscript was drafted by WA. All authors reviewed the draft manuscript and approved the final version.

## Supplementary material

Supplementary Appendix 1 CHWs' survey questionnaire.
