# Supplementary material for: “We just have to help”: Community health workers' informal task-shifting and task-sharing practices for hypertension and diabetes care in Nigeria
Source: Front Public Health. 2023 Jan 26;11:1038062. doi: 10.3389/fpubh.2023.1038062 (PMC9909193; doi:10.3389/fpubh.2023.1038062)
Supplement: Supplementary Appendix 1 — CHWs' survey questionnaire. [file Data_Sheet_1.PDF]

Community Health Workers Survey

The aim of the survey

This survey will be conducted to understand the current role of Community Health Workers ***(Community Health Officers, Community Health Extension Workers, Junior Community Health Extension Workers)*** in the prevention and management non-communicable diseases like cardiovascular diseases and diabetes using a checklist. It has three main sections; socio demographic variables; questions related to trainings, managing and supervision; and the role of Community Health Workers for the delivery of service for non-communicable diseases prevention and management.

A. Socio demographic characteristics

1. Address of Community Health Workers' work place
   a. State____
   b. LGA ____
   c. Ward ____
2. Age ______________ (years)
3. Sex: Male ( ) Female ( )
4. What is your highest educational level? (a) Ordinary National Diploma (b) Higher national Diploma (c) Bachelor degree (d) Master degree (e) Others (please specify) _________________________
5. What is your religion? (a) Christianity (b) Islam (c) Others __________________________
6. What is your marital status? (a) Single (b) Married (c) Divorce/Separated (d) Widow/Widower.
7. What is your family size including you? _______________________________________
8. What is the average monthly household income of your family? (i.e. you and your spouse if married) ____
9. How many years have you worked as a Community Health Worker? _________ (years)
10. How far is the health centre from where you live? _______ (km)
11. How do you get to work from home? (a) Walk (b) Bicycle (c) personal motorbike (d) personal car (e) public transport
12. How far is the health centre from where you live? ____ (kilometres)

13. How long does it take you to travel to the health centre from where you live? ____ (minutes)
14. Does your health centre have regular electricity? (a) Yes (b) No
15. Does your health centre have regular water supply? (a) Yes (b) No

B. Training, managing and supervision of Community Health Workers

16. After completing your training, did you attend any training/workshop related to chronic disease prevention and management e.g. diabetes and hypertension? (a) Yes (b) No
    (If no, go to Q.18)
17. If yes, list the training/workshop and duration
    a. Training__________________________ Duration_______________
    b. Training__________________________ Duration_______________
    c. Training__________________________ Duration_______________
    d. Training__________________________ Duration_______________

18. Does anyone supervises you? (a) Yes (b) No ***(if no, go to Q. 21)***
19. Who supervises you? ____

    ____

    (Mention rank and position of your supervisors)
20. What does your supervisor do when he or she visits you?
    a. Observation of service delivery (a) Yes (b) No
    b. Coaching and skills development (a) Yes (b) No
    c. Problem solving (a) Yes (b) No
    d. Record Review (a) Yes (b) No
    e. Supply check (a) Yes (b) No
    f. Others (please, specify) ____

21. Do you supervise anyone? (a) Yes (b) No ***(if "No", go to Q.24)***
22. Who do you supervise? ____

    ____

(Mention rank and position of your supervisee)

23. What do you do when you visit for supervision?
    a. Observation of service delivery (a) Yes (b) No
    b. Coaching and skills development (a) Yes (b) No
    c. Problem solving (a) Yes (b) No
    d. Record Review (a) Yes (b) No
    e. Supply check (a) Yes (b) No
    f. Others (please, specify) ____
24. Are you satisfied working as a community health worker? (a) Yes (b) No
25. Do you plan to continue as a community health worker in the future? (a) Yes (b) No
26. Which of the following do you think are reasons for attrition (***i.e. leaving the job***) of community health workers?
    a. Inadequate compensation/salary **(a) Yes (b) No**
    b. Low support from the higher administrative bodies **(a) Yes (b) No**
    c. Undermining the role of the community health workers by other professionals (i.e. not being respected or recognised by other health workers) **(a) Yes (b) No**
    d. Low respect from the community **(a) Yes (b) No**
    e. Family reasons **(a) Yes (b) No**
    f. Excess workload **(a) Yes (b) No**
    g. Other (please specify) ____
27. What are some challenges you face while carrying out chronic disease (hypertension and diabetes) prevention and management as a community health worker?
    a. Inadequate training (a) Yes (b) No
    b. Inadequate supervision (a) Yes (b) No
    c. Inadequate supply and equipment (a) Yes (b) No
    d. Low motivation (a) Yes (b) No
    e. Poor infrastructure (a) Yes (b) No
    f. Other (please specify) _____________________________________

C. These are activities listed to be deliver by the Community Health Workers (CHWs) for the delivery of non-communicable diseases prevention and management (Guideline on Minimum Standards for PHC in Nigeria)			
**S.No**	**Main domain of the CHWs activities on NCDs**	**Which one of the following activities are available or are you currently providing for your patients with cardiovascular diseases or diabetes?**	**Category**
28.	Registration of basic demographic and clinical data of people reporting to primary care with major chronic diseases	a. Register adults with Hypertension<br>b. Were you trained for this?	a) Yes<br>b) No<br>a) Yes<br>b) No
		a. Register adults with Diabetes<br>b. Were you trained for this?	a) Yes<br>b) No<br>a) Yes<br>b) No
29.	Early identification of people with non-communicable diseases / opportunistic screening in primary care centers	a. Organized screening for hypertension<br>b. Were you trained for this?	a) Yes<br>b) No<br>a) Yes<br>b) No
		c. Organized screening for diabetics<br>d. Were you trained for this?	a) Yes<br>b) No<br>a) Yes<br>b) No
		e. Organized screening for cancers<br>f. Were you trained for this?	a) Yes<br>b) No<br>a) Yes<br>b) No
30.	Increasing community awareness through community engagement and mobilization	a. Health education on fruits and vegetables intake<br>b. Were you trained for this?	a) Yes<br>b) No<br>a) Yes<br>b) No
		c. create community awareness on regular physical activity 30 minutes daily<br>d. Were you trained for this?	a) Yes<br>b) No<br>a) Yes<br>b) No
		e. create community awareness on reduced salt intake<br>f. Were you trained for this?	a) Yes<br>b) No<br>a) Yes<br>b) No

		g. Create community awareness on tobacco cessation<br><br>h. Were you trained for this?	a) Yes<br>b) No<br><br>a) Yes<br>b) No
		i. create community awareness on weight control<br><br>j. Were you trained for this?	a) Yes<br>b) No<br><br>a) Yes<br>b) No
31.	Application of evidence-based interventions for chronic disease prevention and care services offer by the Community Health Workers	a) Conduct regular home visits to assist patients with non-communicable diseases (diabetes and hypertension)<br><br>b) Were you trained for this?	a) Yes<br>b) No<br><br>a) Yes<br>b) No
		c) Follow patient up to ensure adherence to medications for NCDs<br><br>d) Were you trained for this?	a) Yes<br>b) No<br><br>a) Yes<br>b) No
		e) Blood pressure measurement for monitoring of hypertension management<br><br>f) Were you trained for this?	a) Yes<br>b) No<br><br>a) Yes<br>b) No
		g) Blood glucose measurement for monitoring of diabetics management<br><br>h) Were you trained for this?	a) Yes<br>b) No<br><br>a) Yes<br>b) No
		i) Counseling for<br>i. smoking cessation<br><br>ii. adopting a healthy diet<br><br>iii. weight control<br><br>j) Were you trained for these?	1. Yes<br>2. No<br><br>1. Yes<br>2. No<br><br>1. Yes<br>2. No<br><br>a) Yes<br>b) No
32.	Referral system	a) Counselling and motivation for referral<br><br>b) Were you trained for this?	a) Yes<br>b) No<br><br>a) Yes<br>b) No

		c) Mobilizing support as required from the community (VDC/WDC) to effect referrals<br><br>d) Were you trained for this?<br><br>e) Effecting referral for all cases to the next health facilities<br><br>f) Were you trained for this?	a) Yes<br>b) No<br><br>a) Yes<br>b) No<br><br>a) Yes<br>b) No<br><br>a) Yes<br>b) No
		g) Accompany patients to health facilities<br><br>h) Were you trained for this?	a) Yes<br>b) No<br><br>a) Yes<br>b) No
		i) Get feedback and follow up of the referred patient<br><br>j) Were you trained for this?	a) Yes<br>b) No<br><br>a) Yes<br>b) No
33.	Essential drugs	a) Replenishment of drug stock from LGA and distribution to lower levels<br><br>b) Were you trained for this?	a) Yes<br>b) No<br><br>a) Yes<br>b) No
		c) Prescribe drugs for hypertension or diabetes<br><br>d) Were you trained for this?	a) Yes<br>b) No<br><br>a) Yes<br>b) No
		e) Refill drugs for hypertension or diabetes<br><br>f) Were you trained for this?	a) Yes<br>b) No<br><br>a) Yes<br>b) No
